# Nontypeable *Haemophilus influenzae*-Induced MyD88 Short Expression Is Regulated by Positive IKKβ and CREB Pathways and Negative ERK1/2 Pathway

**DOI:** 10.1371/journal.pone.0144840

**Published:** 2015-12-15

**Authors:** Carla S. Andrews, Masanori Miyata, Seiko Susuki-Miyata, Byung-Cheol Lee, Kensei Komatsu, Jian-Dong Li

**Affiliations:** Center for Inflammation, Immunity & Infection, Institute for Biomedical Sciences, Georgia State University, Atlanta, Georgia, United States of America; Queen's University Belfast, UNITED KINGDOM

## Abstract

Airway diseases such as asthma and chronic obstructive pulmonary disease (COPD) are characterized by excessive inflammation and are exacerbated by nontypeable *Haemophilus influenzae* (NTHi). Airway epithelial cells mount the initial innate immune responses to invading pathogens and thus modulate inflammation. While inflammation is necessary to eliminate a pathogen, excessive inflammation can cause damage to the host tissue. Therefore, the inflammatory response must be tightly regulated and deciphering the signaling pathways involved in this response will enhance our understanding of the regulation of the host inflammatory response. NTHi binds to TLR2 and signal propagation requires the adaptor molecule myeloid differentiation factor 88 (MyD88). An alternative spliced form of MyD88 is called MyD88 short (MyD88s) and has been identified in macrophages and embryonic cell lines as a negative regulator of inflammation. However, the role of MyD88s in NTHi-induced inflammation in airway epithelial cells remains unknown. Here we show that NTHi induces MyD88s expression and MyD88s is a negative regulator of inflammation in airway epithelial cells. We further demonstrate that MyD88s is positively regulated by IKKβ and CREB and negatively regulated by ERK1/2 signaling pathways. Taken together these data indicate that airway inflammation is controlled in a negative feedback manner involving MyD88s and suggest that airway epithelial cells are essential to maintain immune homeostasis.

## Introduction

Airway diseases such as asthma and chronic obstructive pulmonary disease (COPD) affect more than one-half billion people globally. These diseases are characterized by inflammation and are exacerbated by respiratory pathogens [1–3]. Nontypeable *Haemophilus influenzae* (NTHi) is a Gram-negative, non-encapsulated, opportunistic coccobacillus and the most common colonizing bacterium in the nasopharynx microbiome. NTHi is a major cause of airway inflammation during stable and exacerbated states of COPD. In addition to COPD exacerbations, NTHi also causes other respiratory diseases such as chronic bronchitis [4, 5]. Antibiotics are commonly used to treat NTHi infections. However, the increasing numbers of antibiotic resistant strains presents an urgent need for the development of novel non-antibiotic therapeutic [6, 7].

Airway epithelial cells are critically involved in host defense by providing the initial physical barrier and mounting innate immune responses to antigens making these cells essential regulators of airway structure, function and inflammation. Epithelial cells modulate host defense by: producing antimicrobial peptides and growth factors; producing, degrading or inhibiting inflammatory mediators, such as cytokines and chemokines; and recruiting leukocytes. Epithelial cells initially recognize and respond to antigens via pattern recognition receptors (PRRs) called toll-like receptors (TLRs). TLRs then initiate signaling cascades involving kinases and critical adaptor molecules leading to transcriptional upregulation of the genes involved in host defense [8, 9]. NTHi binds to Toll-like receptor 2 (TLR2) on epithelial cells and ultimately results in the translocation of transcription factors into the nucleus leading to transcriptional regulation of genes involved in the inflammatory response and host defense. Subsequent effector mechanisms clear the infection and the inflammatory response is then terminated. While inflammation is essential to eradicate these pathogens, an excessive response may be deleterious to the host [10–12]. Therefore immune homeostasis maintained by epithelial cells requires tight regulation of the inflammatory signaling pathways. Deciphering these signaling pathways will enhance our understanding of the regulation of the host response.

Following TLR2 activation by NTHi recognition, the adaptor protein myeloid differentiation factor 88 (MyD88) is recruited to the receptor. MyD88 then recruits and activates IL-1 receptor-associated kinases (IRAKs). Interactions between the IRAKs and MyD88 lead to the activation of tumor-necrosis factor-receptor-associated factors (TRAFs). Subsequent downstream phosphorylation and ubiquitination events lead to activation of NF-κB. Multiple intracellular molecules that activate NF-κB can also inhibit the inflammatory response induced by TLR2 signaling in a negative feedback manner [13, 14]. MyD88 is a critical bottleneck adaptor protein that is recruited to many TLRs, which recognize a variety of antigens, and therefore must be tightly regulated. This is accomplished by the alternatively spliced short form of MyD88 (MyD88s) [15]. MyD88s transcription is induced by interleukin-1 and lipopolysaccharide and lacks the intermediate domain between the death domain and the Toll/IL-1 receptor (TIR) domain of full length MyD88. MyD88s cannot recruit and phosphorylate the IRAKs and therefore cannot activate NF-κB [16–18]. The role of MyD88s and how it is regulated in airway epithelial cells in response to NTHi remains undefined. An alternative strategy for anti-inflammatory therapeutics is to up-regulate expression of the negative regulators of inflammation. As such, the first step for designing potential therapeutics is to identify those negative regulators, confirm their modulatory role and ascertain the signaling networks and transcription factors that regulate their expression. Therefore, understanding the regulation of MyD88s may aid in the discovery of novel anti-inflammatory therapeutics. Furthermore, MyD88s may prove to be a good therapeutic target for regulating inflammation.

In the present study, we investigated the role of MyD88s in airway epithelial cells. We showed that NTHi induces MyD88s and that MyD88s is a negative regulator of NTHi-induced NF-κB activation and subsequent inflammation. We found that IKKβ and CREB are required for NTHi-induced MyD88s expression. Furthermore, we identified ERK1/2 as negative regulators for MyD88s transcription. Our data provide the initial steps for the design of a novel potential therapeutic target to regulate inflammation and restore immune homeostasis.

## Results

### NTHi Induces MyD88 Short Expression in Airway Epithelial Cells *in vitro* and in the Lung of Mice

**Fig 1:**
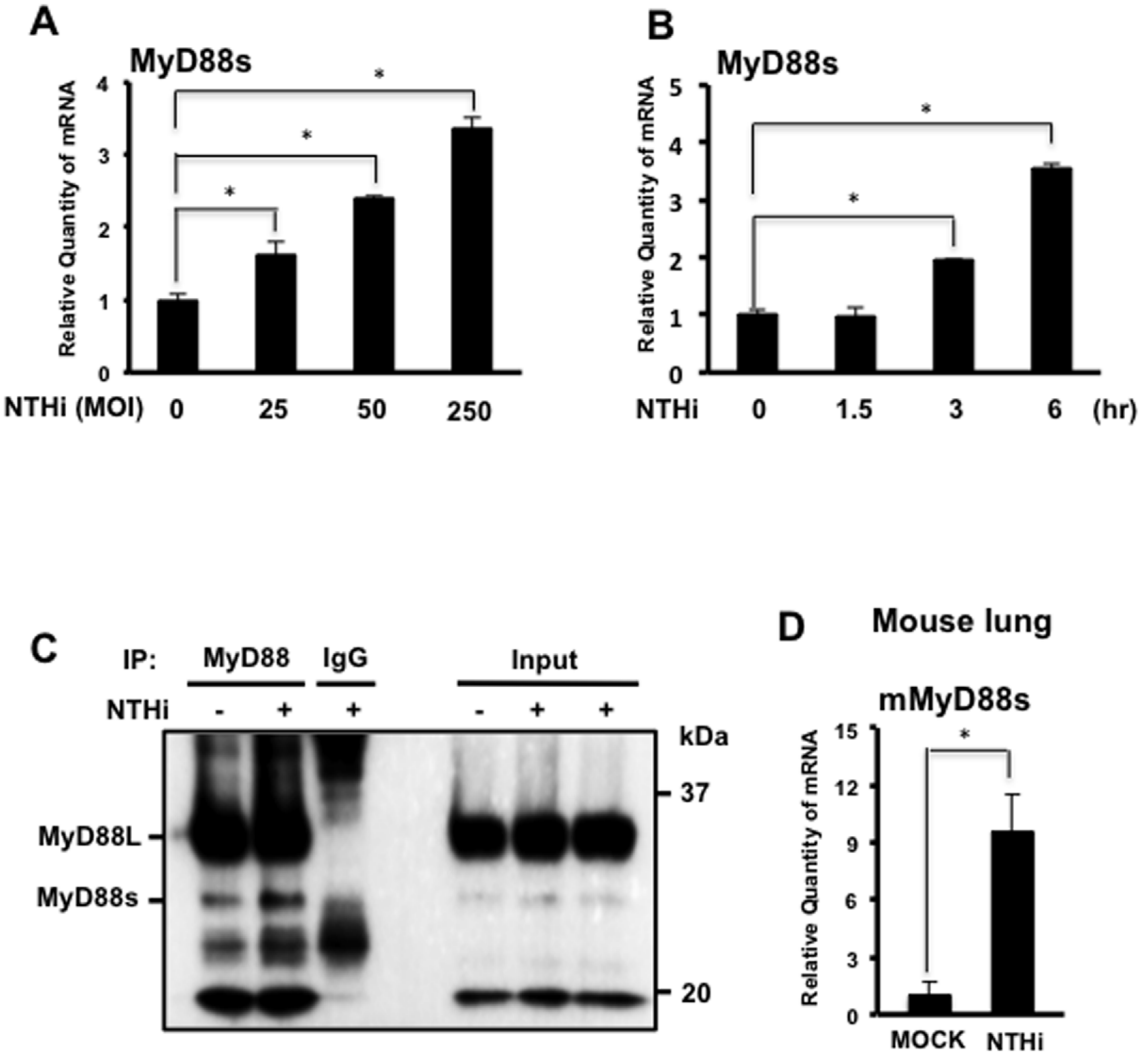
NTHi induces MyD88 short expression in airway epithelial cells and in the lung of mice. **(A)** Human bronchial epithelial BEAS-2B cells were stimulated with increasing doses of NTHi as indicated for 6 hours and the relative quantity of human MyD88 short (MyD88s) mRNA was measured by real-time Q-PCR analysis. **(B)** Cells were stimulated with NTHi at a MOI of 50 for increasing intervals of time as indicated and the relative quantity of MyD88s mRNA was measured by real-time Q-PCR analysis. **(C)** Cells were stimulated with NTHi for 9 hours and MyD88s protein was immunoprecipitated, and then immunoprecipitates and cell lysates were analyzed by western blotting with antibody against MyD88. **(D)** C57BL/6 mice were inoculated with NTHi at a concentration of 5 x 10^7^ CFU. Lung tissue was harvested for total mRNA extraction and the relative quantity of mouse MyD88s mRNA was measured by real-time Q-PCR analysis. Data are mean ± SD (*n* = 3). *p<0.*05*. Statistical analysis was performed using Student’s *t-*test. Data are representative of three or more independent experiments.

Previous studies showed that MyD88s transcription is induced by interleukin-1 and lipopolysaccharide [16]. To determine if NTHi induces MyD88s expression in airway epithelial cells, MyD88s mRNA was measured using real-time quantitative PCR (Q-PCR) analysis. NTHi induced MyD88s mRNA expression in a dose- (Fig 1A) and time-dependent (Fig 1B) manner in BEAS-2B cells. Furthermore, we also confirmed that NTHi induced MyD88s protein expression in BEAS-2B cells by performing immunoprecipitation and western blot analysis (Fig 1C). Similar to the *in vitro* findings, NTHi induced upregulation of MyD88s at the mRNA level in the mouse lung as assessed by Q-PCR analysis (Fig 1D).

### MyD88 Short is a Negative Regulator of NTHi-induced Inflammation

**Fig 2:**
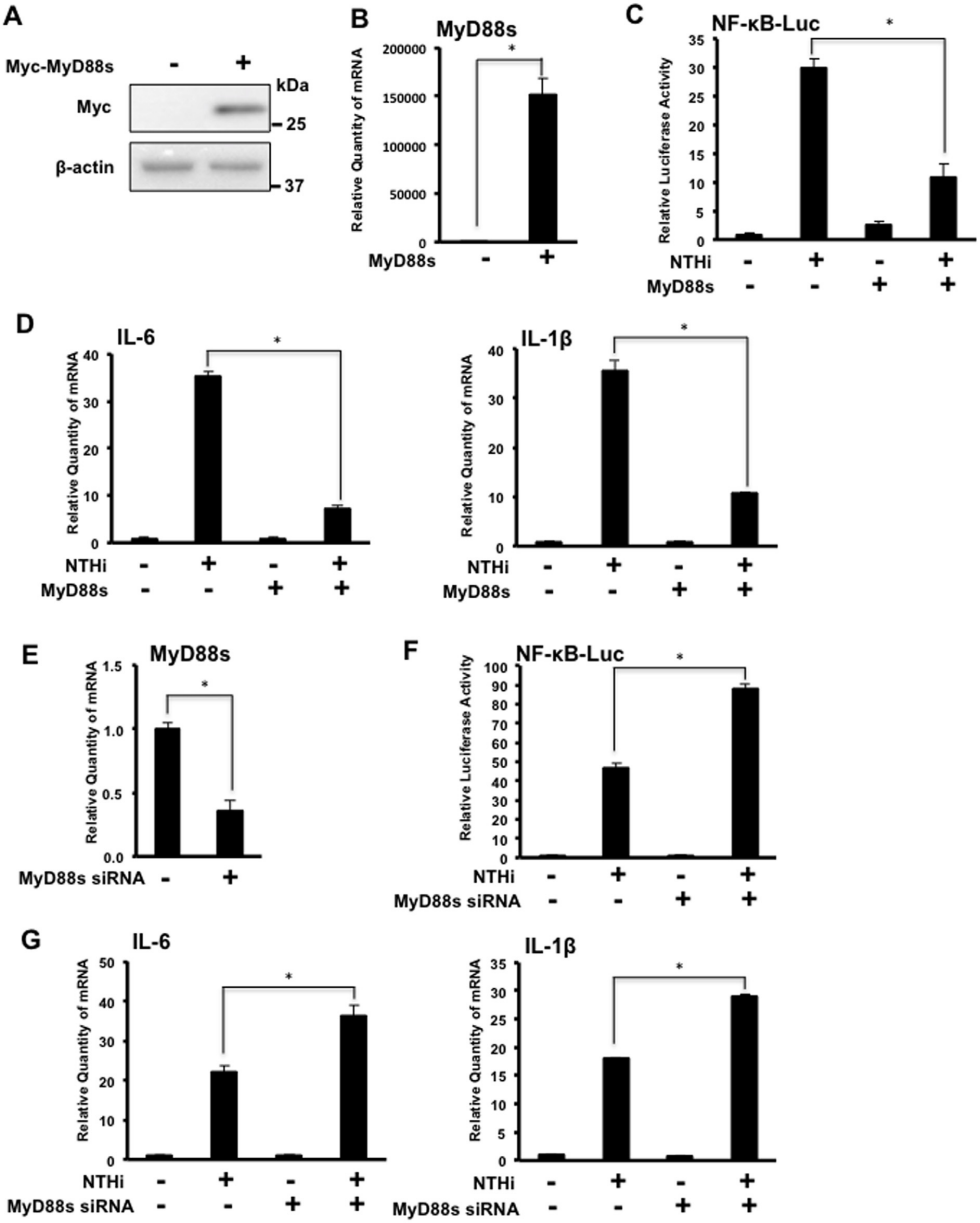
MyD88 short is a negative regulator of NTHi-induced inflammation in airway epithelial cells. **(A-B)** BEAS-2B cells were transfected with a Myc-tagged MyD88s overexpression plasmid or empty vector and (**A**) MyD88 protein was visualized via western blot analysis using antibodies against Myc and (**B**) Relative quantity of human MyD88s mRNA was measured by real-time Q-PCR analysis. **(C-D)** Cells were transfected with the MyD88s overexpression plasmid alone or together with a NF-κB luciferase reporter construct followed by NTHi stimulation for 6 hours. (**C**) Relative NF-κB promoter activity was measured. (**D**) Relative quantities of human IL-6 or human IL-1β mRNA were measured by real-time Q-PCR analysis. **(E)** Cells were transfected with MyD88s siRNA or control siRNA and relative quantity of MyD88s mRNA was measured by real-time Q-PCR analysis. **(F-G)** Cells were transfected with the MyD88s siRNA or control siRNA alone or together with a NF-κB luciferase reporter construct followed by NTHi stimulation for 6 hours. **(F)** Relative NF-κB promoter activity was measured. **(G)** Relative quantities of IL-6 or IL-1β mRNA were measured by real-time Q-PCR analysis. Data are mean ± SD (*n* = 3). *p<0.*05*. Statistical analysis was performed using Student’s *t-*test. Data are representative of three or more independent experiments.

Previous studies indicate that MyD88s acts as a negative regulator for NF-κB signaling pathway [18]. To investigate the functional role of MyD88s in NTHi-induced inflammation in airway epithelial cells, BEAS-2B cells were transfected with a MyD88s overexpression plasmid (Fig 2A–2D) or MyD88s siRNA (Fig 2E–2G) alone or together with a NF-κB luciferase promoter construct and stimulated with NTHi. MyD88s overexpression significantly reduced NTHi-induced NF-κB luciferase activity (Fig 2C). MyD88s overexpression also decreased NTHi-induced expression of pro-inflammatory cytokines IL-6 and IL-1β (Fig 2D). MyD88s siRNA significantly enhanced NTHi-induced NF-κB luciferase activity (Fig 2F), and increased NTHi-induced expression of IL-6 and IL-1β (Fig 2G). Together these results show that MyD88s is a negative regulator of NTHi-induced inflammation.

### IKKβ is Required for NTHi-induced MyD88 Short Expression in Airway Epithelial Cells

**Fig 3:**
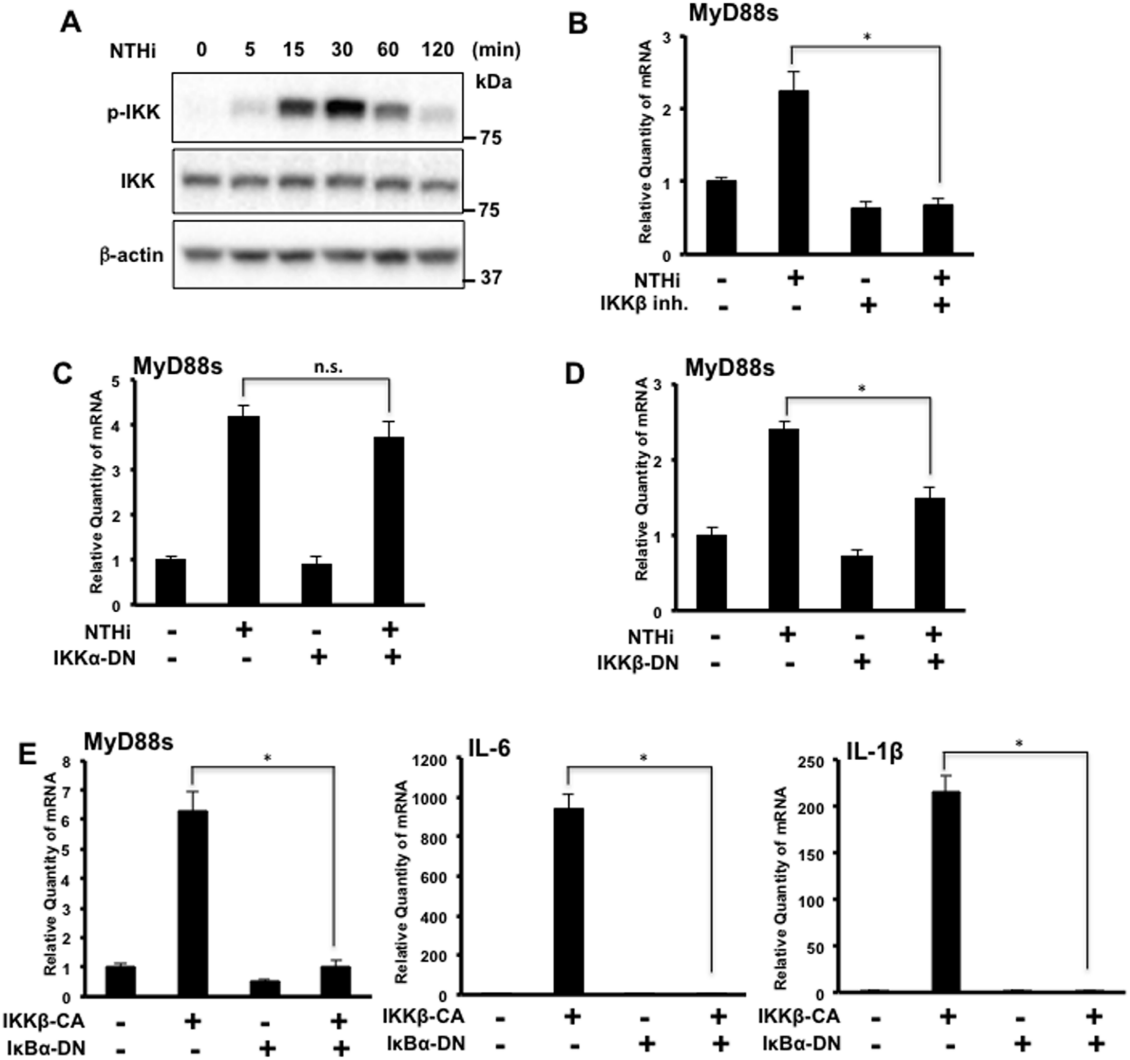
IKKβ is required for NTHi-induced MyD88 short expression in airway epithelial cells. **(A)** BEAS-2B cells were stimulated with NTHi at various intervals of time as indicated. Phospho-IKK, total IKK and β-actin were visualized via western blot analysis. **(B)** Cells were treated with IKKβ inhibitor (1 μM) prior to NTHi stimulation for 6 hours and relative quantity of human MyD88s mRNA was measured by real-time Q-PCR analysis. **(C-E)** Cells were transfected with (**C**) IKKα-DN, (**D**) IKKβ-DN or (**E**) cotransfected with IκBα-DN and IKKβ-CA. Following NTHi stimulation for 6 hours, the relative quantities of MyD88s mRNA, human IL-6 mRNA and human IL-1β were measured via real-time Q-PCR analysis. Data are mean ± SD (*n* = 3). *p<0.*05*. Statistical analysis was performed using Student’s *t-*test. n.s., nonsignificant. Data are representative of three or more independent experiments.

Because NTHi-mediated NF-κB activation is controlled by IKKα/β [19], we first confirmed that NTHi induces peak IKKα/β phosphorylation in BEAS-2B cells at 30 minutes post stimulation (Fig 3A). To determine if IKKβ is required for NTHi-induced MyD88s expression, BEAS-2B cells were treated with an IKKβ inhibitor prior to NTHi stimulation. IKKβ inhibition significantly decreased NTHi-induced MyD88s mRNA expression (Fig 3B). To elucidate the predominate IKK isoform involved in NTHi-induced MyD88s mRNA expression, BEAS-2B cells were transfected with an IKKα or an IKKβ dominant-negative (DN) mutant form and stimulated with NTHi. The IKKβ DN mutant decreased NTHi-induced MyD88s mRNA expressions significantly while the IKKα mutant did not significantly alter MyD88s mRNA expression (Fig 3C and 3D). Since NTHi-mediated IKKβ phosphorylation induces phosphorylation of IκBα [19], BEAS-2B cells were cotransfected with an IκBα DN mutant and/or an IKKβ constitutively active (CA) form. Cotransfected cells showed a significant reduction in MyD88s, IL-6 and IL-1β mRNA expression compared to cells transfected with the IKKβ CA alone (Fig 3E). Together these data show that IKKβ signaling is required for NTHi-induced MyD88s mRNA expression in airway epithelial cells.

### CREB is Required for NTHi-induced MyD88 Short Expression in Airway Epithelial Cells

**Fig 4:**
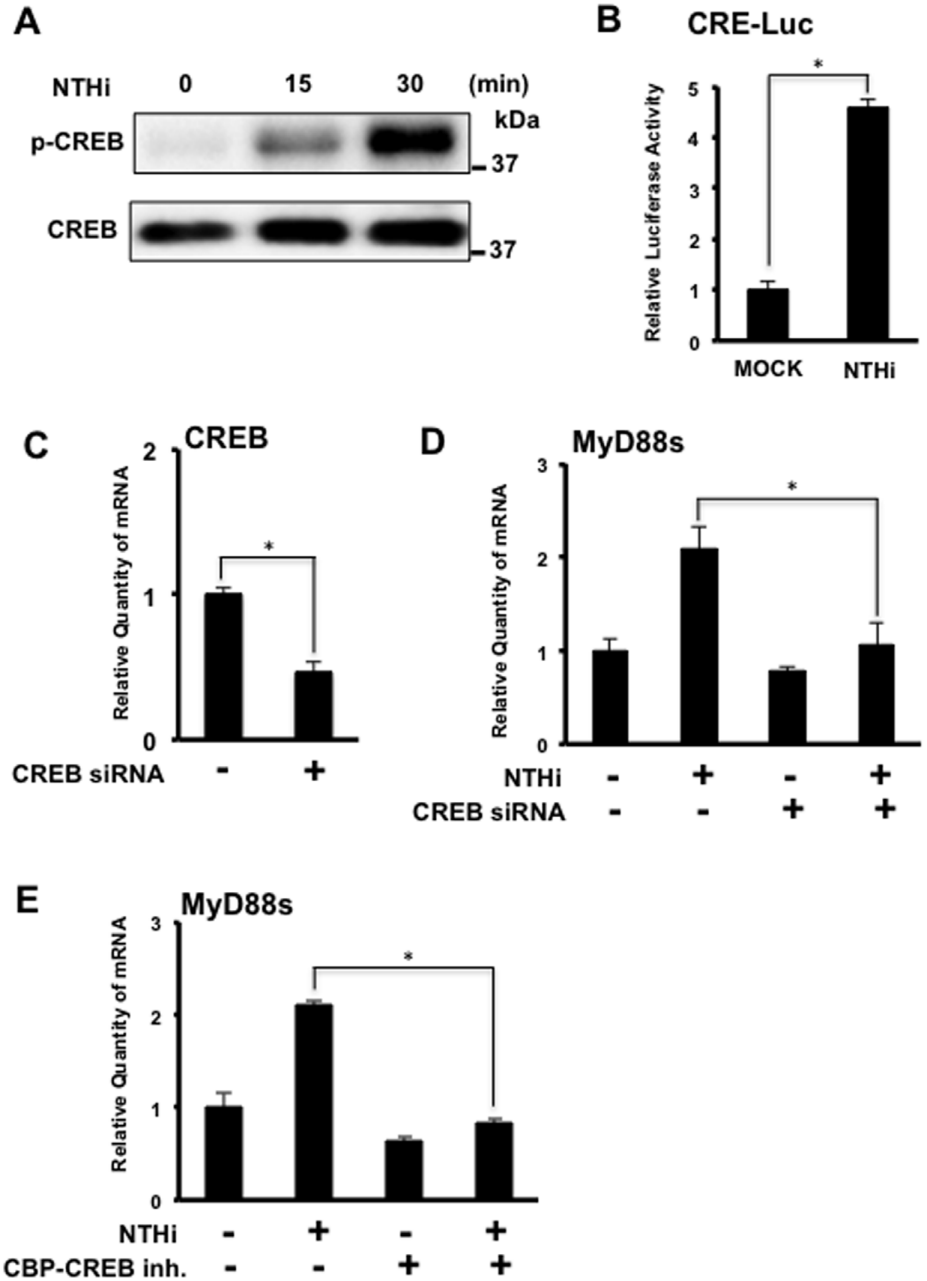
CREB is required for NTHi-induced MyD88 short expression in airway epithelial cells. **(A)** BEAS-2B cells were stimulated with NTHi at various time intervals as indicated. Phospho-CREB and total CREB were visualized via western blot analysis. **(B)** Cells were transfected with the CRE-luciferase construct and stimulated with NTHi for 6 hours. Relative luciferase activity was measured. **(C-E)** Cells were (**C-D**) transfected with CREB siRNA or control siRNA or (**E**) treated with CBP-CREB inhibitor (100 μM) prior to NTHi stimulation for 6 hours. Relative quantity of human MyD88s mRNA was measured via real-time Q-PCR analysis. Data are mean ± SD (*n* = 3). *p<0.*05*. Statistical analysis was performed using Student’s *t-*test. Data are representative of three or more independent experiments.

Numerous studies have linked the transcription factor cAMP-response element binding protein (CREB) to modulation of various inflammatory mediators [20–22]. Furthermore, TLR2 stimulation has been shown to phosphorylate CREB, which is crucial for CREB mediated gene transcription, and NTHi activates TLR2 [11, 23, 24]. To determine if NTHi induces CREB phosphorylation, BEAS-2B cells were stimulated with NTHi. Marked CREB phosphorylation was observed at 30 minutes (Fig 4A). To determine if NTHi activates CREB-mediated transcription, cells were transfected with a CREB response element (CRE) luciferase construct. NTHi significantly induced CRE luciferase activity (Fig 4B). Cells transfected with CREB siRNA also showed a significant reduction in NTHi-induced MyD88s mRNA expression (Fig 4C and 4D). Additionally, cells treated with a CBP-CREB inhibitor also showed a significant decrease in NTHi-induced MyD88s mRNA expression (Fig 4E). Together these data show that CREB is required for NTHi-induced MyD88s expression in airway epithelial cells.

### ERK1/2 are Negative Regulators of NTHi-induced MyD88 Short Expression in Airway Epithelial Cells

**Fig 5:**
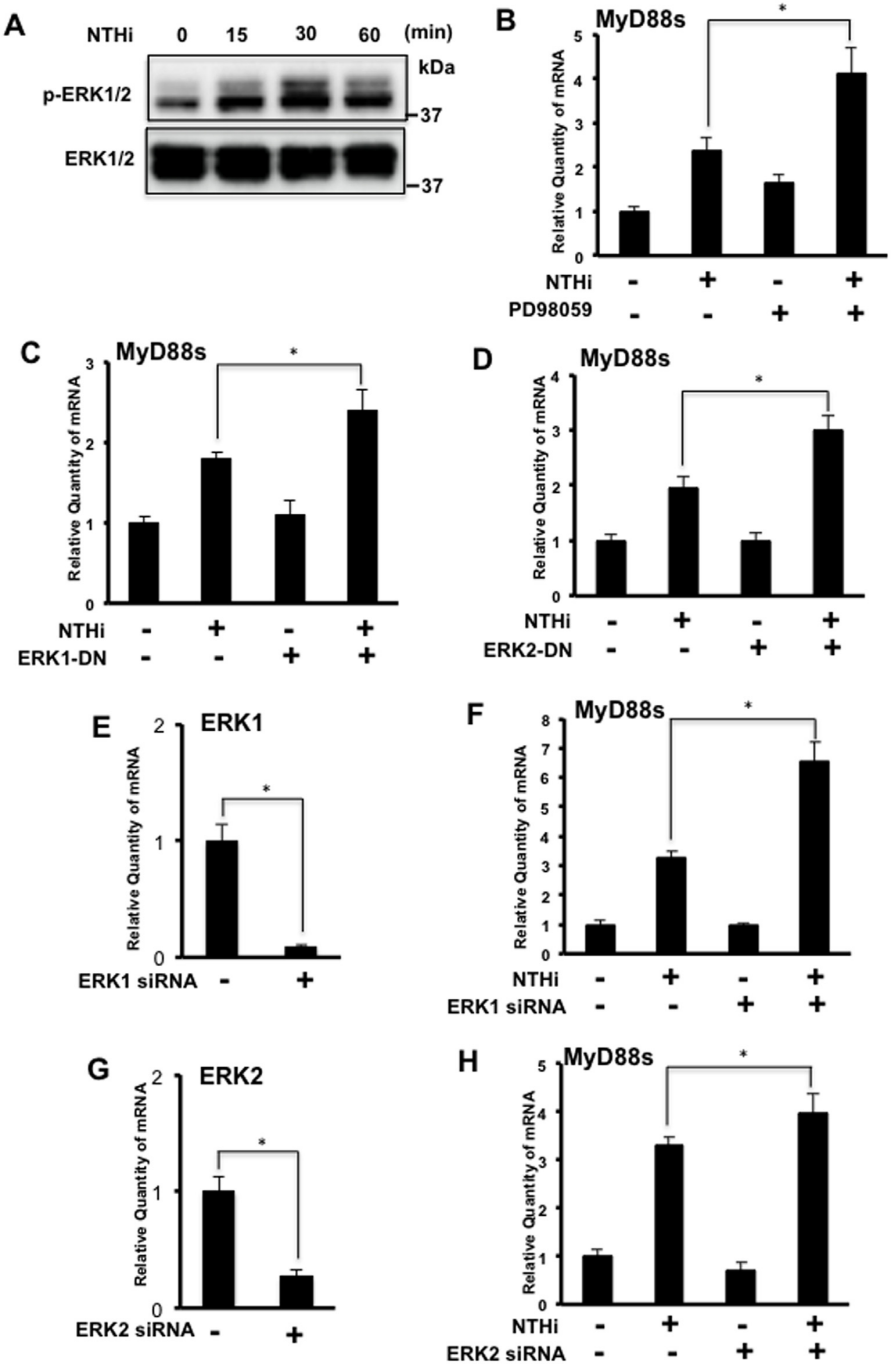
ERK 1/2 is a negative regulator for NTHi-induced MyD88 short expression in airway epithelial cells. **(A)** BEAS-2B cells were stimulated with NTHi at various time intervals as indicated. Phospho-ERK and total ERK were visualized via western blot analysis. **(B)** Cells were treated with PD98059 (10 μM) followed by NTHi stimulation for 6 hours. The relative quantity of human MyD88s was measured by real-time Q-PCR analysis. **(C-H)** Cells were transfected with (**C**) ERK1-DN, (**D**) ERK2-DN, (**E-F**) ERK1 siRNA or (**G-H**) ERK2 siRNA. Following NTHi stimulation for 6 hours, the relative quantity of MyD88s was measured by real-time Q-PCR analysis. Data are mean ± SD (*n* = 3). *p<0.*05*. Statistical analysis was performed using Student’s *t-*test. Data are representative of three or more independent experiments.

The ERK signaling pathways have been shown to play an important role in controlling inflammatory responses [25, 26]. We thus sought to determine the roles of ERK1/2 in NTHi-induced MyD88s expression. First we confirmed that NTHi induced peak phosphorylation of ERK1 and ERK2 at 30 minutes in BEAS-2B cells (Fig 5A). Next, BEAS-2B cells were treated with PD98059, an upstream ERK inhibitor, prior to NTHi stimulation. Interestingly, inhibition of ERK increased NTHi-induced MyD88s mRNA expression (Fig 5B). To confirm the role of ERK, cells were transfected with ERK1 and ERK2 DN mutants and both enhanced NTHi-induced MyD88s mRNA expression (Fig 5C and 5D). Additionally, ERK1 (Fig 5E and 5F) and ERK2 (Fig 5G and 5H) siRNA both enhanced NTHi-induced MyD88s mRNA expression. Together these data show that ERK1/2 are negative regulators for NTHi-induced MyD88s expression in airway epithelial cells.

## Discussion

Airway diseases, such as asthma and COPD are characterized by inflammation and are exacerbated by respiratory pathogens, namely NTHi [1–3]. While inflammation is a necessary response to clear infections, an overactive inflammatory response may be harmful to the host. Therefore, the signaling pathways activated by pathogen recognition must be tightly regulated. In this study, we showed that NTHi induces expression of MyD88s in airway epithelial cells and in the lung of mice. We also found that MyD88s is a negative regulator of NTHi-induced inflammation. Further, we demonstrated that IKKβ and CREB are required for NTHi-induced MyD88s expression. Additionally, we found that while NTHi activates the ERK signaling pathway, ERK1/2 negatively regulate NTHi-induced MyD88s expression. Our study demonstrates for the first time the role of MyD88s in regulating inflammation in airway epithelial cells and the signaling pathways that regulate the transcription of NTHi-induced MyD88s expression.

Of particular interest is our novel finding that NTHi induces MyD88s expression (Fig 1) and MyD88s is a negative regulator of inflammation in airway epithelial cells (Fig 2). MyD88s is a smaller splice variant of MyD88 that lacks exon 2 and was originally identified in mouse macrophages [16]. Subsequent studies found that MyD88s can be induced by LPS and IL-1 but cannot activate NF-κB in HEK293T cells, macrophages and other embryonic cell lines [16–18]. A later study found that monocytes isolated from septic patients had increased MyD88s mRNA expression compared to healthy controls suggesting an attempt to restore immune homeostasis [27]. Epithelial cells are critical regulators of immune homeostasis as they are the first cells to encounter an invading pathogen. Therefore, these data demonstrate the significant role that airway epithelial cells play in the tight regulation of inflammation and immune homeostasis.

**Fig 6:**
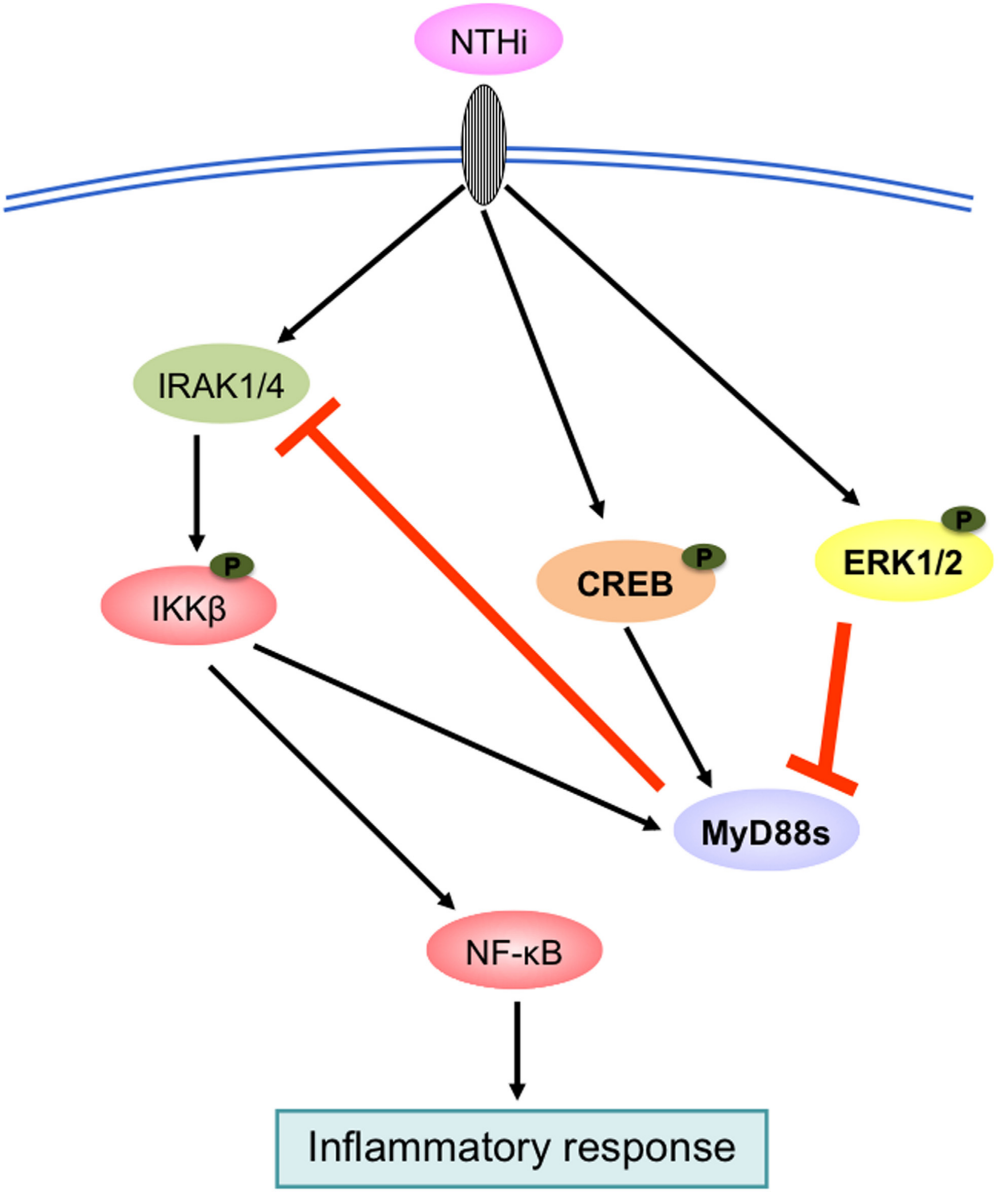
Schematic model illustrating NTHi-induced MyD88s is regulated by IKKβ, CREB and ERK1/2 signaling pathways. As indicated NTHi-induced MyD88s expression is positively regulated via IKKβ and CREB and negatively regulated via ERK1/2 creating a negative feedback loop to maintain immune homeostasis.

We also investigated the signaling pathways that regulate NTHi-induced MyD88s expression. NF-κB is a major family of transcription factors that controls expression of many inflammatory mediators. Two key upstream molecules participating in the regulation of NF-κB activation are the IκB kinases IKKα and IKKβ [19, 28, 29]. We show that NTHi-induced MyD88s expression is predominantly positively regulated by IKKβ (Fig 3). Cells co-transfected with IKKβ CA and IκBα DN showed a decrease in expression of MyD88s, IL-6 and IL-1β expression compared to cells transfected with IKKβ CA alone. Since MyD88s targets upstream of IKKβ at the IRAKs, cytokine expression induced by IKKβ-CA would not be affected MyD88s expression. Additionally, we examined the role of the transcription factor CREB in modulating MyD88s expression because it has been shown to be involved in regulating pro- and anti-inflammatory molecules [20–22]. Our data show that CREB positively regulates NTHi-induced MyD88s expression (Fig 4). Lastly, we explored the role of ERK1/2 in the regulation of MyD88s. ERK1/2 play an important role in the inflammatory response and NTHi phosphorylates and activates ERK1/2 [25, 26]. We found that ERK1/2 are negative regulators of NTHi-induced MyD88s expression in airway epithelial cells (Fig 5). Collectively these data identify the positive and negative regulatory signaling pathways that control MyD88s expression in epithelial cells (Fig 6).

In conclusion, this study demonstrates that NTHi induces expression of the negative regulator of inflammation MyD88s and elucidates the signaling pathways involved in the regulation of MyD88s expression in airway epithelial cells. Inflammation is a crucial response to eliminating an infection and the long form of MyD88 is an essential bottleneck adaptor protein that many TLRs rely upon to propagate signaling pathways [8, 9, 13, 15]. However, excessive inflammation can be deleterious to the host [10–12]. Therefore, the inflammatory response must be tightly regulated. TLR2 signaling initiated by NTHi results in a robust inflammatory response and MyD88s induced by NTHi results in a negative feedback manner. Deciphering the signaling pathways that control the regulatory molecules of inflammation is critical for developing effective therapeutics. Future studies may focus on therapeutics that target the negative regulators of inflammation.

## Materials and Methods

### Cell Culture

Human bronchial epithelial cells BEAS-2B cells (ATCC) were maintained in RPMI 1640 medium (Gibco) supplemented with 10% (v/v) heat-inactivated FBS (Sigma-Aldrich). Cells were cultured at 37°C in a humidified atmosphere of 5% CO_2_ with a passage number no greater than 25 [30, 31].

### Bacteria Strain and Culture Conditions

A clinical isolate of NTHi strain 12 was grown on chocolate agar plates at 37°C in 5% CO_2_ overnight, harvested and incubated overnight in brain heart infusion (BHI) broth supplemented with 3.5 μg ml^-1^ NAD and hemin. Bacteria were subcultured in fresh BHI broth to log phase growth, as measured by optical density, pelleted, washed, and resuspended in DMEM for *in vitro* experiments or isotonic saline for *in vivo* experiments. The cells were stimulated with NTHi at a multiplicity of infection (MOI) of 50 unless otherwise specified for 6 hours or as indicated.

### Reagents and Antibodies

The IKKβ inhibitor IKK-2 inhibitor IV was purchased from Calbiochem. PD98059 was purchased from Enzo Life Sciences. CBP-CREB interaction inhibitor was purchased from EMD Millipore. All inhibitors were reconstituted in dimethyl sulfoxide (DMSO) and diluted with RPMI 1640 media to a final DMSO concentration of 0.1%. DMSO in RPMI 1640 was used as a control. Cells were treated with 1 μM of the IKKβ inhibitor, 10 μM of PD98059 or 100 μM of the CBP-CREB interaction inhibitor in a final volume of 500 μL per well of 12 well plates. Antibodies against MyD88, Myc, phospho-IKKα/β, total IKKα/β, phospho-CREB, total CREB, phospho-ERK1/2, and total ERK1/2 were purchased from Cell Signaling Technology. Antibody against β-actin was purchased from Santa Cruz Biotechnology. Cells were treated with or without IKK inhibitor, PD98059 or CBP-CREB interaction inhibitor for 1 hour prior to stimulation with NTHi.

### Plasmids, Transfections, and Luciferase Reporter Assay

The expression plasmids IKKα-dominant negative (DN), IKKβ-DN, IκBα-DN, a constitutively active form of IKKβ (IKKβ-CA), ERK1-DN, ERK2-DN and the NF-κB luciferase reporter construct have been previously described [19, 32]. The CREB response element (CRE)-luciferase reporter construct was purchased from Promega. A full length MyD88 overexpression plasmid was previously subcloned by PCR from pCMV-HA-MyD88 (Addgene) into a pcDNA3-Myc vector. The MyD88s overexpression plasmid was constructed by deletion of exon 2 of the MyD88 plasmid with QuickChange II Site-Directed Mutagenesis kit (Agilent Technologies) with the following primers: sense 5’-CTGGAGCTGGGACCCAGCATTGGGCATATGCCTGAGCGTTTC-3’; antisense 5’-GAAACGCTCAGGCATATGCCCAATGCTGGGTCCCAGCTCCAG-3’. All transient transfections were performed in triplicate using TransIT-LT-1 reagent (Mirus) according to the manufacturer’s instructions. An empty vector was used as a control in all experiments. Cells were seeded in 12 well plates and at 80% confluence transfected with 0.2 μg of plasmid per well and/or 0.1 μg of the NF-κB luciferase construct per well or 0.3 μg of the CRE-luciferase construct per well, together with pSV-β-Galactosidase control vector (Promega). Twenty-four hours after transfection, cells were stimulated with or without NTHi. Cell lysate was used for mRNA analysis, western blot analysis or to measure transcriptional activity of NF-κB and CRE luciferase activity. Luciferase activity was determined using Luciferase Assay System (Promega), and β-Galactosidase activity was measured using β-Galactosidase Enzyme Assay System (Promega). Luciferase activity was normalized with respect to β-galactosidase activity.

### Small Interfering RNA (siRNA)

siRNA for MyD88s, 5’-CCCAGCATTGGGCATATGCCT-3’, and the SMARTpool siRNA for CREB, ERK1, ERK2 and ON-TARGETplus non-targeting control pool were purchased from Dharmacon. BEAS-2B cells were seeded in 12 well plates and at 80% confluence, transfected with 20 nM MyD88s siRNA, 20 nM ERK siRNA or 50 nM CREB siRNA and 1 μL of DharmaFECT (Dharmacon) in 1000 μL of media per well according to the manufacturer’s instructions alone or together with the NF-κB luciferase reporter construct using Lipofectamine 3000 (Invitrogen) following the manufacturer’s instructions. Forty-eight hours after transfection, cells were stimulated with or without NTHi. Cell lysate was used for mRNA analysis or to measure transcriptional activity of NF-κB using the luciferase assay.

### RNA Isolation and Real-time Quantitative RT-PCR (Q-PCR)

Q-PCR analysis of human and mouse MyD88s, human IL-6, and human IL-1β was conducted as follows. Total RNA was isolated with TRIzol reagent (Invitrogen) according to the manufacturer’s instructions. Reverse transcription was performed using 1 μg of RNA and TaqMan reverse transcription reagents; 10x RT buffer, MgCl_2_, dNTPs, random hexamers, oligo (dT) primers, RNase inhibitor and reverse transcriptase (Applied Biosystems). The reaction was performed for 10 minutes at 25°C followed by 60 minutes at 42°C. SYBR Green Universal Master Mix (Applied Biosystems) was used for the PCR amplification. In brief, reactions were performed in triplicate containing 2 x universal master mix, 1 μL of template cDNA, 500 nM primers in a final volume of 12.5 μL, and they were analyzed in a 96-well optical reaction plate (Applied Biosystems). An ABI 7500 sequence detector with accompanying software (Applied Biosystems) was used for amplification and quantification. The comparative threshold cycle (Ct) method was used to obtain relative quantities of mRNAs that were normalized using human cyclophilin A or mouse glyceraldehyde-3-phosphate dehydrogenase (GAPDH) as an endogenous control. The primer sequences for human cyclophilin A, IL-6 and IL-1β, as well as mouse GAPDH were described previously [30, 31]. The primer sequences for human CREB, ERK1, ERK2, MyD88s, as well as mouse MyD88s are as follows: human CREB forward primer, 5’-GAACCAGCAGAGTGGAGATG-3’; human CREB reverse primer, 5’-GGCATAGATACCTGGGCTAATG-3’; human ERK1 forward primer, 5’-GCTGAACTCCAAGGGCTATAC-3’; human ERK1 reverse primer, 5’-GTTGAGCTGATCCAGGTAGTG-3’; human ERK2 forward primer, 5’-TTCAGTGCACCTACTGCTTAC-3’; human ERK2 reverse primer, 5’- CAGCAGGGCATCATGTAGAA-3’; human MyD88s forward primer, 5’-GACCCAGCATTGGGCATATG-3; human MyD88s reverse primer, 5’-CGGTCAGACACACACAACTTC-3’; mouse MyD88s forward primer, 5’-GGAGCTGAAGTCGCGCATCGGACAAAC-3’; mouse MyD88s reverse primer, 5’-GTCTGTTCTAGTTGCCGGATCATCTCCTG-3’.

### Western Blot Analysis and Immunoprecipitation

Western blot analyses were performed as previously described [33]. Following transfection and/or NTHi stimulation, cells were lysed, incubated on ice for 30 minutes and centrifuged at 12,000 x *g* for 15 minutes. Supernatants were separated on a 10% SDS-PAGE gel, transferred to polyvinylidene fluoride membrane. The membranes were blocked with a solution of Tris-buffered saline (TBS) containing 0.1% Tween 20 (TBS-T) and 5% nonfat dry milk, then incubated with primary antibodies against MyD88, Myc, β-actin phospho-IKKα/β, total IKKα/β, phospho-CREB, total CREB, phospho-ERK1/2 or total ERK1/2 at a 1:2000 dilution in 5% BSA-TBS-T over night. After three washes in TBS-T, the membrane was incubated with secondary HRP-conjugated rabbit or mouse IgG antibody (Cell Signaling) at a 1:5000 dilution in 5% nonfat dry milk-TBS-T for 1 hour. Proteins were visualized using Amersham ECL Prime Western Blotting Detection Reagent (GE Healthcare Biosciences). For immunoprecipitation, cell lysates were immunoprecipitated with 4 μL of antibodies against MyD88 or normal IgG (Santa Cruz Biotechnology) overnight at 4°C and then conjugated to protein G Plus agarose beads (Santa Cruz Biotechnology) for 2 hours at 4°C.

### Mice and Animal Experiments

C57BL/6 mice were purchased form the National Cancer Institute, National Institutes of Health. Mice were intratracheally inoculated with NTHi at a concentration of 5 x 10^7^ CFU per mouse or saline as control. Mice were sacrificed 6 hours after bacterial inoculation and lung tissue was harvested for total RNA extraction as previously described [34]. All animal experiments were approved by the Institutional Animal Care and Use Committee at Georgia State University.

### Statistical Analysis

All experiments were repeated in at least three independent experiments. Data are shown as the mean ± SD of *n* determinations. Statistical evaluation was done by unpaired Student’s *t*-test, and *p < 0*.*05* was taken as a significant difference.
